# Source of Circulating Pentraxin 3 in Septic Shock Patients

**DOI:** 10.3389/fimmu.2018.03048

**Published:** 2019-01-04

**Authors:** Chloé Albert Vega, Marine Mommert, Mathilde Boccard, Thomas Rimmelé, Fabienne Venet, Alexandre Pachot, Veronique Leray, Guillaume Monneret, Benjamin Delwarde, Karen Brengel-Pesce, François Mallet, Sophie Trouillet-Assant

**Affiliations:** ^1^Joint Research Unit Hospices Civils de Lyon-bioMérieux, Hospices Civils de Lyon Centre Hospitalier Lyon Sud, Lyon, France; ^2^Medical Diagnostic Discovery Department (MD3), bioMérieux S.A., Pierre Bénite, France; ^3^Département des Maladies Infectieuses et tropicales, Hospices Civils de Lyon, Lyon, France; ^4^EA 7426 Pathophysiology of Injury-Induced Immunosuppression, PI3, Claude Bernard Lyon 1 University-bioMérieux-Hospices Civils de Lyon, Edouard Herriot Hospital, Lyon, France; ^5^Anesthesia and Critical Care Medicine Department, Hospices Civils de Lyon, Edouard Herriot Hospital, Lyon, France; ^6^Hospices Civils de Lyon, Edouard Herriot Hospital, Immunology Laboratory, Lyon, France; ^7^Faculté de Médecine Lyon Est, Virpath - Université Lyon, CIRI, INSERM U1111, CNRS 5308, ENS, UCBL, Lyon, France

**Keywords:** pentraxin 3, sepsis, septic shock, immune dysfunction, endotoxin tolerance

## Abstract

Sepsis, which is the leading cause of death in intensive care units (ICU), has been acknowledged as a global health priority by the WHO in 2017. Identification of biomarkers allowing early stratification and recognition of patients at higher risk of death is crucial. One promising biomarker candidate is pentraxin-3 (PTX3); initially elevated and persistently increased plasma concentration in septic patients has been associated with increased mortality. PTX3 is an acute phase protein mainly stored in neutrophil granules. These cells are responsible for rapid and prompt release of PTX3 in inflammatory context, but the cellular origin responsible for successive days' elevation in sepsis remains unknown. Upon inflammatory stimulation, PTX3 can also be produced by other cell types, including endothelial and immune cells. As in septic patients immune alterations have been described, we therefore sought to investigate whether such cells participated in the elevation of PTX3 over the first days after septic shock onset. To address this point, PTX3 was measured in plasma from septic shock patients at day 3 after ICU admission as well as in healthy volunteers (HV), and the capacity of whole blood cells to secrete PTX3 after inflammatory stimulation was evaluated *ex vivo*. A significantly mean higher (100-fold) concentration of plasma PTX3 was found in patients compared to HV, which was likely due to the inflammation-induced initial release of the pre-existing PTX3 reservoir contained in neutrophils. Strikingly, when whole blood was stimulated *ex vivo* with LPS no significant difference between patients and HV in PTX3 release was found. This was in contrast with TNFα which decreased production was illustrative of the endotoxin tolerance phenomenon occurring in septic patients. Then, the release of PTX3 protein from a HV neutrophil-free PBMC endotoxin tolerance model was investigated. At the transcriptional level, PTX3 seems to be a weakly tolerizable gene similar to TNFα. Conversely, increased protein levels observed in anergy condition reflects a non-tolerizable phenotype, more likely to an anti-inflammatory marker. Hence, altered immune cells still have the ability to produce PTX3 in response to an inflammatory trigger, and therefore circulating white blood cell subset could be responsible of the sustained PTX3 plasma levels over the first days of sepsis setting.

## Introduction

There are 31.5 million cases of sepsis per year that lead to 6 million deaths and 3 million people suffer from impairments leading to post-sepsis hospital re-admission (1). The official definition of sepsis is a life-threatening organ dysfunction caused by a dysregulated host response to infection (2) and, although infection is the initial trigger of sepsis response, the dysregulated immune response remains even after successful treatment of the infection (3). Due to the high heterogeneity among patients, early stratification and recognition of patients at higher risk of death is of importance as timely and appropriate decisions as to the best therapeutic approach is crucial to improve survival and decrease in-hospital mortality rates. In this context, several circulating biomarkers have been investigated in clinical studies and pentraxin 3 (PTX3) shows promising performance.

Pentraxin 3 is an acute phase protein which belongs to the long pentraxin subfamily, conserved in evolution, which acts as a key component of humoral innate immunity in microbial infections. Evidence suggests that PTX3 is a key homeostatic component at the crossroad of innate immunity, inflammation, tissue repair, and cancer (4). PTX3 binds conserved microbial structures and self-components under conditions of inflammation and activates effector functions (5). It has been found to be secreted by multiple cells including immune, vascular, lymphatic, endothelial, and epithelial cells (4). Neo-synthesis of PTX3 in these cells, except for neutrophils, is strongly induced by cytokines such as IL-1, TNF-α and by TLR agonists, but not by IL-6 or interferons (6). In neutrophils, PTX3 is synthetized in the bone marrow and stored in neutrophil granules, co-localized with lactoferrin (secondary granules) in physiological conditions, ready to be release upon inflammatory signals (7). Once released into the circulation, neutrophils lose the ability to produce PTX3 mRNA (8). The very high blood PTX3 levels observed as soon as onset of an injury is related to the release of preformed PTX3 contained in neutrophils. This was clearly described by Maugeri et al. who reported that neutrophils were responsible for plasma PTX3 concentration elevation within 6 h from onset of clinical symptoms of acute myocardial infarction, and that this returned to normal values 48 h (9).

PTX3 is barely detectable in the plasma of healthy individuals (<2 ng/mL), but its concentration can increase to up to 100 ng/ml during sepsis depending on the severity of disease (10). In septic shock patients, early high plasma PTX3 predicts subsequent new organ failure, and a smaller subsequent drop in circulating PTX3 over time predicts an increased risk of death (11, 12). Nevertheless, it is known that during septic shock immune cells are impaired (13); septic patients have markedly increased numbers of circulating neutrophils of various degrees of maturation with disrupted functions including impaired phagocytosis, reduced reactive oxygen species (ROS) production, and loss of chemotactic activity (14). Moreover, such patients have greater proportion of immature neutrophils with decreased levels of intracellular lactoferrin (15). Neutrophils may therefore not be the only source of PTX3 during sepsis. We then sought to investigate whether other immune cells participated in the elevation of PXT3 over the first days after septic shock onset. In the present study, using whole blood of sepsis patients and healthy volunteers (HV) as well as an *in vitro* model that mimics immune alterations found in patients, we examined the transcriptomic and proteomic changes of PTX3 upon *ex vivo* stimulation challenge.

## Materials and Methods

### Study Population

This clinical study was conducted on septic shock patients admitted to the intensive care unit (ICU) of the Edouard Herriot Hospital (Hospices Civils de Lyon, Lyon, France) and is part of a wider study on ICU-induced immune dysfunctions. It was approved by the regional ethics committee (*Comité de Protection des Personnes Sud-Est II*, number 11236), which waived the need for written informed consent because the study was observational with a low risk for the patients and no specific procedure other than routine blood sampling was required. This study is also registered at the French ministry of research (*Ministère de l'Enseignement supérieur, de la Recherche et de l'Innovation*; DC-2008-509) and at the national data protection commission (*Commission Nationale de l'Informatique et des Libertés*). Oral information and non-opposition to inclusion in the study were mandatory and recorded in patients' clinical files.

Patients with septic shock were included prospectively. Septic shock was defined according to the Society of Critical Care Medicine and the European Society of Intensive Care Medicine (2): vasopressor requirement to maintain a mean arterial pressure of 65 mmHg or greater and serum lactate level >2 mmol/L (>18 mg/dL) in the absence of hypovolemia. The exclusion criteria were age < 18 years, the presence of aplasia or immunosuppressive disease (e.g., HIV infection). At admission, data collected included demographic characteristics (age, gender), admission category (elective or emergency surgery, medicine) and site of primary infection; two clinical scores were recorded: the initial severity assessed by the Simplified Acute Physiology Score (SAPS II; range: 0–194) at admission and the Sequential Organ Failure Assessment (SOFA) score (range: 0–24), 24 h after ICU stay. Laboratory data during follow-up was also collected, as was death during the ICU stay. In addition, study-specific experiments were performed (see details below) on routine blood samples (EDTA-coated tubes and heparin-coated tubes) taken at day 3–4 after septic shock onset.

Concomitantly, EDTA- and heparin-coated blood tubes from HV aged ≥50 years were obtained from the national blood service (*Etablissement Français du Sang*, EFS) and used immediately. According to EFS standardized procedures for blood donation and to provisions of the articles R.1243–49 and following ones of the French public health code, a written non-opposition to the use of donated blood for research purposes was obtained from HV. The blood donors' personal data were anonymised before transfer to our research laboratory. We obtained the favorable notice of the local ethical committee (Comité de Protection des Personnes Sud-Est II, Bâtiment Pinel, 59 Boulevard Pinel, 69,500 Bron) and the acceptance of the French ministry of research (*Ministère de l'Enseignement supérieur, de la Recherche et de l'Innovation*, DC-2008-64) for handling and conservation of these samples.

### Biological Samples and *in vitro* Experiment

#### Plasma

Plasma samples were obtained after collection of whole blood in EDTA-coated tubes from 30 patients at day 3–4 after ICU admission and from 10 HV and were frozen until batch analysis.

#### TruCulture® Stimulation

Heparinized-whole blood (1 mL), collected at the same time-point than plasma samples, from patients and HV was distributed into prewarmed TruCulture® tubes (Myriad Rbm, Austin, TX, USA) containing the medium alone (Null) or the medium with lipopolysaccharide (LPS; 100 ng/mL). These were then inserted into a dry block incubator and maintained at 37°C for 24 h. For the kinetic study, whole blood from 3 HV and 3 patients were incubated in TruCulture® tubes at 37°C, and 750 μL were collected for analysis at 1, 2, 4, and 24 h of incubation. Following incubation, the supernatant (media and plasma) and the cellular pellet were collected using a separation valve, according to manufacturer's instructions. Supernatants were aliquoted and immediately frozen at −20°C until batch quantification. Cell pellets were resuspended in 2 mL TRI Reagent® LS (Sigma-Aldrich, Deisenhofen, Germany), vortexed for 2 min, and rested for 10 min at room temperature before −80°C storage.

#### Endotoxin Tolerance Model

Peripheral blood mononuclear cells (PBMCs) were isolated from heparinised venous blood of HV by Ficoll density gradient centrifugation (Eurobio Ingen, Courtaboeuf, France) and washed with PBS. Cells were cultured in 24-well plates at 2.10^6^ cells/mL in *X-Vivo* 20 Medium (Lonza, Verviers, Belgium). PBMCs were first cultured without or with 2 ng/mL LPS (mix of *Escherichia coli* O111:B4, O55:B5, and O127:B8; Sigma-Aldrich) to induce the LPS-primed state and incubated overnight at 37°C and 5% CO_2_. In both conditions, LPS-primed (endotoxin tolerance condition) and unprimed (inflammation condition), PBMCs were incubated a second time for 4 h with 100 ng/mL LPS. A non-stimulated well was used as the control. For each condition, supernatants were collected and stored at −20°C. Cells were harvested, lysed in RLT buffer and stored at −80°C until further processing. The protocol was adapted from Allantaz-Frager et al. (16, 17).

#### Monocyte Cell Line

THP1-Xblue™-MD2-CD14 cells (a human acute monocytic leukemia cell line stably expressing MD2/CD14 genes; Invivogen, San Diego, CA, USA) were cultured in 24-well plates at 1.10^6^ cells/mL in RPMI 1640 medium containing 2 mM L-glutamine, 25 mM HEPES (Thermofisher Scientific, Waltham, MA, USA), 10% heat-inactivated fetal bovine serum (FBS; Eurobio Ingen), 100 μg/mL Normocin (Invivogen) and 100 U/mL-100 μg-mL Pen-Strep (Thermofisher Scientific) (18). Endotoxin tolerance model set up on this cell line was above described.

### mRNA Decay and Intracellular Protein Stability Analysis

The endotoxin tolerance model, above described, performed on the THP1-Xblue™-MD2-CD14 cells was used to evaluate mRNA half-life and intracellular protein stability of PTX3 and TNFα. LPS-primed (endotoxin tolerance condition) and unprimed (inflammation condition) monocytes-like cells were incubated a second time, for 2 h, with LPS (100 ng/mL). Then, actinomycin D (5 μg/mL) or cycloheximide (10 μg/mL) were added to inhibit further transcription and translation, respectively, to both LPS-primed and LPS-unprimed conditions. The incubations were stopped at 0.5, 1, 2, 4, and 6 h for analysis. For untreated and actinomycing conditions, cells were harvested for molecular analysis. For untreated and cycloheximide condition, cells were harvested and solubilized in 200 μL lysis buffer (Human MxA protein ELISA, BioVendor, Brno, Czech Republic) dedicated to intracellular protein ELISA quantitation.

### Protein Analysis

Defective TNF-α production *ex-vivo* is a major trait of sepsis-induced immunosuppression (19), so this pro-inflammatory cytokine was evaluated as reference of PTX3 behavior. PTX3 and TNFα proteins from patients and HV in plasma and Truculture® supernatant were quantified using ELLA nanofluidic system (Biotechne, Minneapolis, MI, USA), according to the manufacturers' instructions. PTX3 and TNFα proteins after lysis of cellular pellets from untreated and cycloheximide conditions in the intracellular protein stability assay were also quantified using ELLA. Intracellular protein levels were expressed as a percentage of the maximal protein level.

These two proteins concentrations, from endotoxin tolerance model in PBMC and in THP1-Xblue™-MD2-CD14 cell culture supernatants, were detected using commercially-available ELISA kits Human TNF-alpha DuoSet ELISA and Human Pentraxin 3/TSG-14 DuoSet ELISA (R&D Systems, Biotechne), in accordance with the manufacturers' instructions. Results are expressed in pg/ml.

### Molecular Detection

For Truculture® cell pellet handling and RNA processing and detection, the protocol was followed according to Urrutia et al. study (20). Cell pellets from Truculture® stimulations kept in TRI Reagen® LS (Sigma-Aldrich) were thawed under agitation. Before processing, thawed samples were centrifuged (3,000 g for 5 min at 4°C) to pellet cellular debris generated during the Trizol lysis. For extraction, a modified protocol of the NucleoSpin 96 RNA tissue kit (Macherey-Nagel Gmbh&Co. KG, Düren, Germany) was followed using a vacuum system. Briefly, 600 μL of clarified Trizol lysate was transferred to a tube preloaded with 900 μL 100% ethanol. The binding mixture was transferred to a silica column, then washed with buffers MW1 and MW2, and RNA was eluted using 30 μL RNase-free water. NanoString technology was used for mRNA detection, a hybridization-based multiplex assay characterized by its amplification-free step; 300 ng of RNA were hybridized to the probes (Supplementary Table 1) at 67°C for 18 h using a thermocycler (Biometra, Tprofesssional TRIO, Analytik Jena AG, Jena, Germany). After removal of excessive probes, samples were loaded into the nCounter Prep Station (NanoString Technologies, Seattle, WA, USA) for purification and immobilization onto the internal surface of a sample cartridge for 2–3 h. The sample cartridge was then transferred and imaged on the nCounter Digital Analyzer (NanoString Technologies) where color codes were counted and tabulated for PTX3 (NM_002852.3) and TNFα (NM_000594.2) genes. Counts number were normalized by the geometric mean of HPRT1 (NM_000194.1), DECR1 (NM_001359.1) and TBP (NM_001172085.1) housekeeping genes count number, as well as the negative and positive control values using nSolver analysis software (version 4.0, Nanostring technologies). Results are expressed in counts and fold change induction.

For endotoxin tolerance model, the protocol followed, with minor modifications, for PBMC and THP1-Xblue™-MD2-CD14 cell pellet handling, RNA processing and detection, was previously described (17). PTX3 and TNFα mRNA from endotoxin tolerance model in PBMC and in THP1-Xblue™-MD2-CD14 cells, but also from untreated and actimnomycin D conditions in the mRNA decay assay were quantified after RNA extraction from cellular pellet. RNeasy plus mini kit (Qiagen, Hilden, Germany) was used for total RNA extraction and RNA quantity was determined using Nanodrop (Thermofisher Scientific), according to the manufacturer's instructions. For mRNA detection, RNA was retro-transcribed using SuperScript VILO cDNA Synthesis kit (Thermofisher Scientific) followed by qPCR, performed using commercial Taqman probes for *TNF?* and *PTX3* (Invitrogen, Carlsbad, CA, USA) and normalized using the *PPIB* housekeeping gene. In the mRNA decay assay, mRNA levels were expressed as a percentage of the maximal mRNA level.

### mHLA-DR Measurement by Flow Cytometry

Circulating monocyte HLA-DR expression (mHLA-DR) from patients was assessed at day 3–4 on peripheral whole blood collected in EDTA anticoagulant tubes by flow cytometry (NAVIOS; Beckman-Coulter, Brea, CA, USA) as previously described (21). Results are expressed as the number of antibodies bound per cells (AB/C). Immunocompetence levels of mHLA-DR are defined as >15,000 AB/c and severe immunoparalysis as >5,000 AB/c (22, 23).

### Statistical Analysis

Results are expressed as median and interquartile ranges [IQR] for continuous variables. Non-parametric data were analyzed using the Mann–Whitney U test. Wilcoxon matched-pairs signed rank test was used for the analysis of THP1-MD2-CD14 experiments. Spearman test was used for correlation analysis. Statistical analyses were conducted using GraphPad Prism® software (version 5; GraphPad software, La Jolla, CA, USA). A *p*-value 0.05 was considered as statistically significant.

## Results

### Clinical Characteristics of the Patients and Healthy Volunteers

From June 2017 to June 2018, 30 patients with septic shock were included (Table 1). The mean age was 65 (range: 19–86) years and 70% were male. The three major sites of infection were abdominal (30%), urinary tract infection (UTI) (13%) and skin and soft tissues (SST) (13%). The mean (range) SOFA score at day 1–2 was 9 (4–15) and SAPS II was 62 (26–93), indicating a high level of severity. 90% of patients had low mHLA-DR at 3–4 days after onset of shock, ranging from moderate to severe immunoparalysis. During ICU stay, mortality was 17% (*n* = 5) and the mean ICU stay among those who were discharged alive was 10 days. Concomitantly, a total of 10 HV were included, 50% were male and the mean age was 54.2 (range: 50–60) years.

**Table 1:** Clinical and immunological data for patients with septic shock.

	**Patients at D3-D4 (*n* = 30)**
Age, years (range)	65 (19–86)
Sex, male, n (%)	21 (70)
SOFA score (range)	9 (4–15)
SAPS II (range)	62 (26–93)
Missing data	3
mHLA-DR (AB/c)	8240 (1644–32790)
**TYPE OF ADMISSION**, ***N*** **(%)**	
Medical	13 (43)
Emergency surgery	17 (57)
**SITE OF PRIMARY INFECTION**, ***N*** **(%)**	
Abdominal	9 (30)
UTI	4 (13)
SST	4 (13)
Other	13 (44)
LOS in the ICU, days (range)	10 (2–34)
Death in the ICU, *n* (%)	5 (17)

*Numeric variables are presented as mean (range). Categorical variables are presented as number of cases, and percentages among the total of patients in parentheses. Simplified Acute Physiology Score (SAPS II) was calculated after admission and Sequential Organ Failure Assessment (SOFA) score was measured after 24 h of ICU stay. mHLA-DR expressed as numbers of anti-HLA-DR antibodies bound per monocyte (AB/C). LOS, length of stay; UTI, urinary tract infection; SST, skin and soft tissue*.

### Plasma PTX3 Concentration

**Figure 1:**
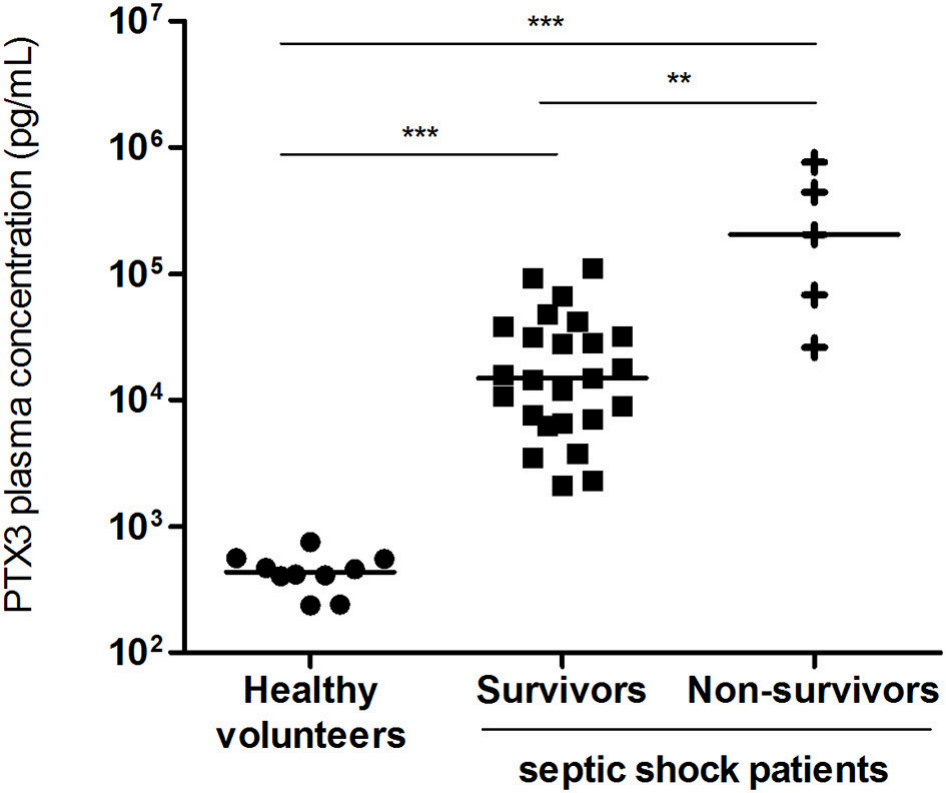
Plasma PTX3 concentration. PTX3 plasma concentration in survivors (*n* = 25) and non-survivors (*n* = 5) septic shock patients, at day 3–4 after ICU admission and in healthy volunteers (*n* = 10). Bar represents the median. **p < 0.001; ***p < 0.001.

The median [IQR] PTX3 plasma levels at day 3–4 after ICU admission was significantly greater in septic shock patients (22031 [7518–52891] pg/mL) than in HV (438 [364–557] pg/mL, *p* < 0.0001; Figure 1). The 5 patients who died during ICU stay had significant higher PTX3 plasma concentration (204879 [47199–604280] pg/mL) compared to those who were discharged alive (14893 [6832–35336] pg/mL, *p* < 0.05; Figure 1). The correlation of plasma PTX3 levels with the degree of immunosuppression of the cells measured on the same day was explored, and no significant correlation was found (r spearman: −0.317, Figure S1).

### *Ex vivo* Whole-Blood LPS Stimulation

**Figure 2:**
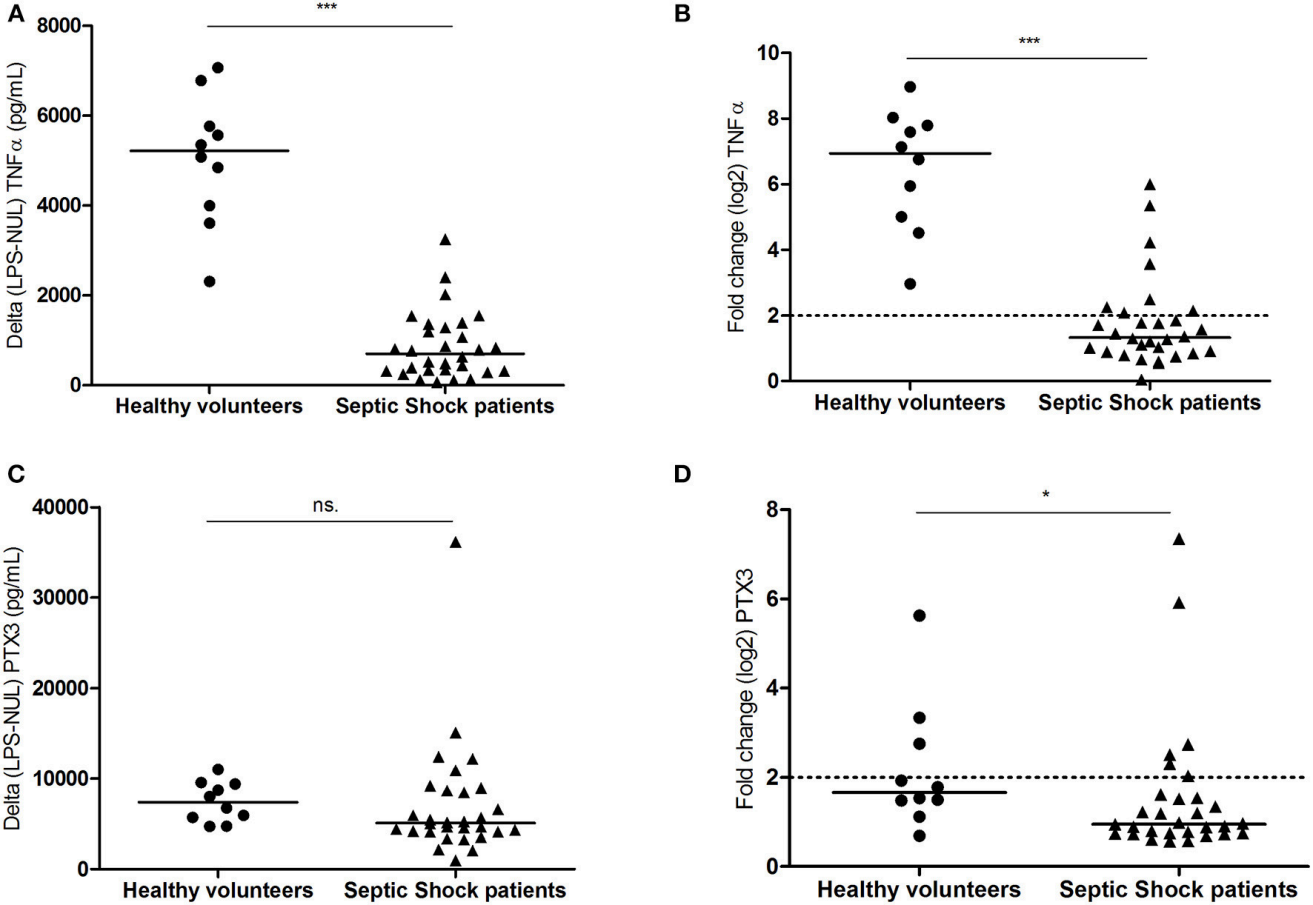
*Ex vivo* whole-blood LPS-stimulation. Whole blood from 10 healthy volunteers and 30 septic shock patients stimulated *ex vivo* with LPS in Truculture® tubes for 24 h. Protein levels **(A–C)** and mRNA gene expression **(B–D)** are plotted for TNFα and PTX3. For cytokine quantification, results were expressed as the difference in cytokines production between LPS stimulated condition and unstimulated conditions (pg/mL). Counts number for mRNA gene expression, were normalized by the geometric mean of *hprt1, decr1*, and *tbp* housekeeping genes. The relative differential expression between LPS-stimulated and unstimulated condition was represented in the figure. Bar represents median. NS: not significant; *p < 0.05; ***p < 0.0001.

After 24 h incubation, we evaluated the *ex vivo* capacity of patient blood cells to express PTX3 and TNFα at the protein and molecular level upon LPS stimulation compared to an unstimulated condition. Due to the high level of PTX3 in septic patients at baseline (unstimulated condition, median [IQR]: 11128 [5052–21559] pg/mL), results were expressed as the difference in cytokine production between the LPS-stimulated condition and the unstimulated condition. Likewise, as PTX3 mRNA counts were higher in patients at baseline than in HV (unstimulated condition, median [IQR]: 233 [75–561] counts in patients and median [IQR]: 59 [38–98] counts in HV, *p* < 0.05), results were expressed as the ratio in counts between the LPS-stimulated condition and the unstimulated condition. Septic shock patients had a significantly attenuated secretion of LPS-stimulated TNFα concentrations (median [IQR]: 701 [314–1301] pg/mL) compared to HV (median [IQR]: 5215 [3895–6014] pg/mL, *p* < 0.0001; Figure 2A). There was a corresponding 5-fold decrease in TNFα gene expression after LPS stimulation in septic shock patients compared to HV (fold change: 1.33 vs. 6.94 respectively, *p* < 0.0001; Figure 2B). Conversely, septic shock patients did not have altered capacity to secrete PTX3 after LPS stimulation. There was no significant difference (Figure 2C, *p* = 0.08) observed in PTX3 concentrations between patients (median [IQR]: 5,077 pg/mL [4128–8737] pg/mL) and HV (median [IQR]: 7393 [5464–9439] pg/mL). At the molecular level, PTX3 gene expression (Figure 2D) by blood cells after LPS stimulation did not seem to be affected in patients (fold change: 0.95). Furthermore, LPS did not induce a significant gene expression of PTX3 in HV (fold change: 1.66).

### mRNA and Protein Kinetic on Whole Blood After LPS-Stimulation

**Figure 3:**
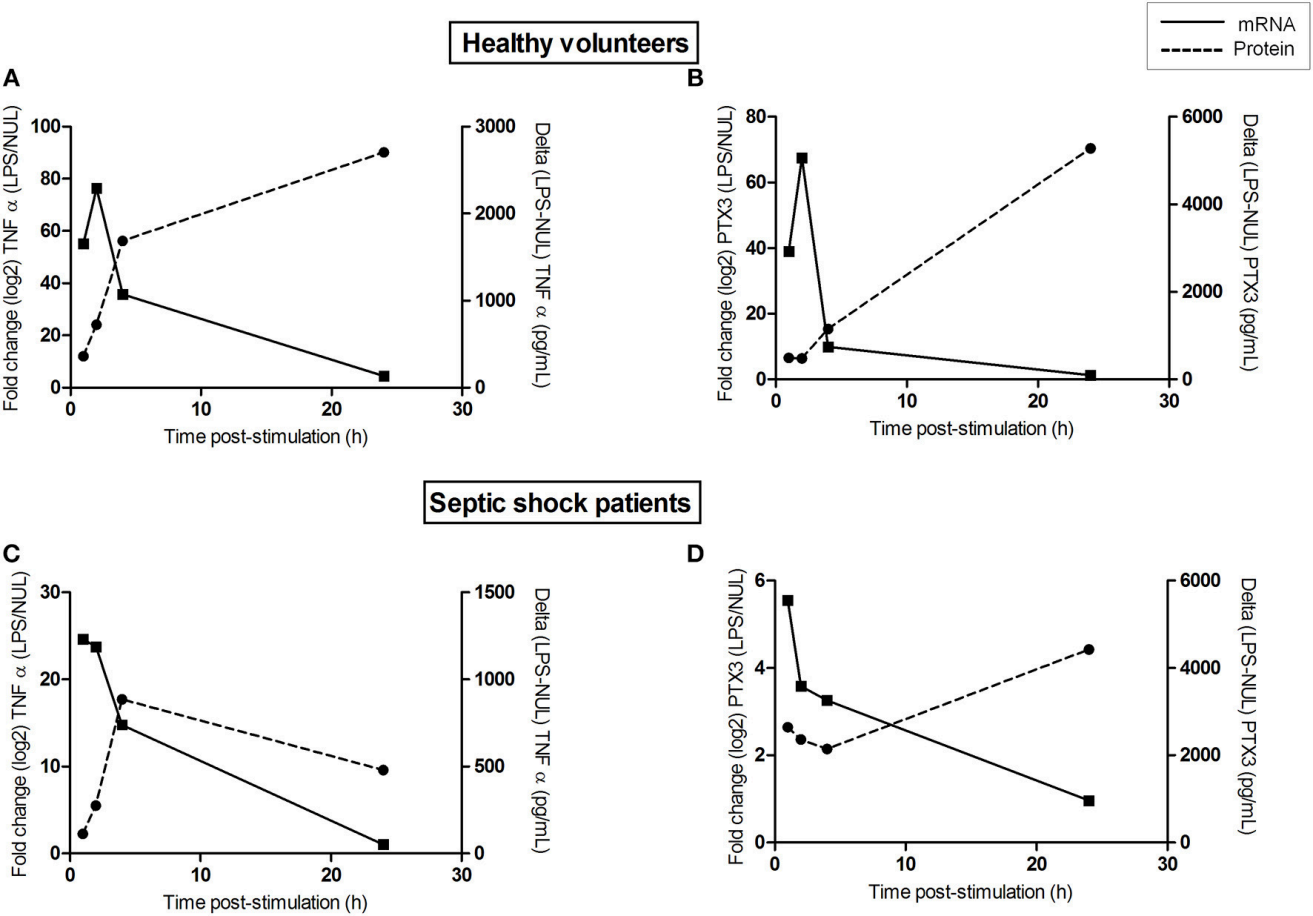
mRNA and protein kinetic on whole blood after LPS-stimulation. Whole blood from 3 healthy volunteers **(A,B)** and 3 septic shock patients **(C,D)** stimulated *ex vivo* with LPS in Truculture® tubes for 1, 2, 4, and 24 h. Protein levels (dashed line) and mRNA gene expression (solid line) are plotted for TNFα **(A,C)** and PTX3 **(B,D)**. For cytokine quantification, results were expressed as the difference in cytokines production between LPS stimulated condition and unstimulated conditions (pg/mL, y right axis). Counts number for mRNA gene expression, were normalized by the geometric mean of *hprt1, decr1*, and *tbp* housekeeping genes. The relative differential expression between LPS-stimulated and unstimulated condition was represented in the figure (y left axis). Lines represents median of the three observations.

Kinetics studies were performed to decipher the apparently unusual dichotomy between PTX3 mRNA expression and protein secretion, in contrast to TNFα. *Ex vivo* whole blood from 3 healthy volunteers and 3 septic shock patients were stimulated with LPS and four time-points were analyzed post-stimulation: 1, 2, 4, and 24 h. In HV, we observed a massive early TNFα mRNA expression at 2 h post-stimulation (80-fold induction) which decreased by half 2 h later and reached low but significant levels of induction 24 h post-stimulation. Protein levels quickly raised 4 h post-stimulation to reach the highest 24 h post-stimulation (Figure 3A). For PTX3 mRNA, the peak expression was also observed 2 h post-stimulation (70-fold), and sharply decreased 2 h later until under significant levels 24 h post-stimulation. PTX3 protein required 24 h to reach high levels post-stimulation (Figure 3B), evidencing a time-lag between transcription and protein secretion. For septic shock patients, an overall decrease in induction efficiency was observed for both TNFα and PTX3 at the transcriptional level, the lowest level being reached after 24 h of stimulation, similarly to HV. Conversely, the highest level of TNFα protein was observed at the earliest time-points, to further decrease until very low levels(Figure 3C), while PTX3 protein levels were elevated during the first 4 h, to reach highest levels 24 h post-stimulation (Figure 3D), similar to 24-h HV levels.

### *In vitro* PBMC- and THP1-MD2-CD14 Cell Line-Based Endotoxin Tolerance Model

**Figure 4:**
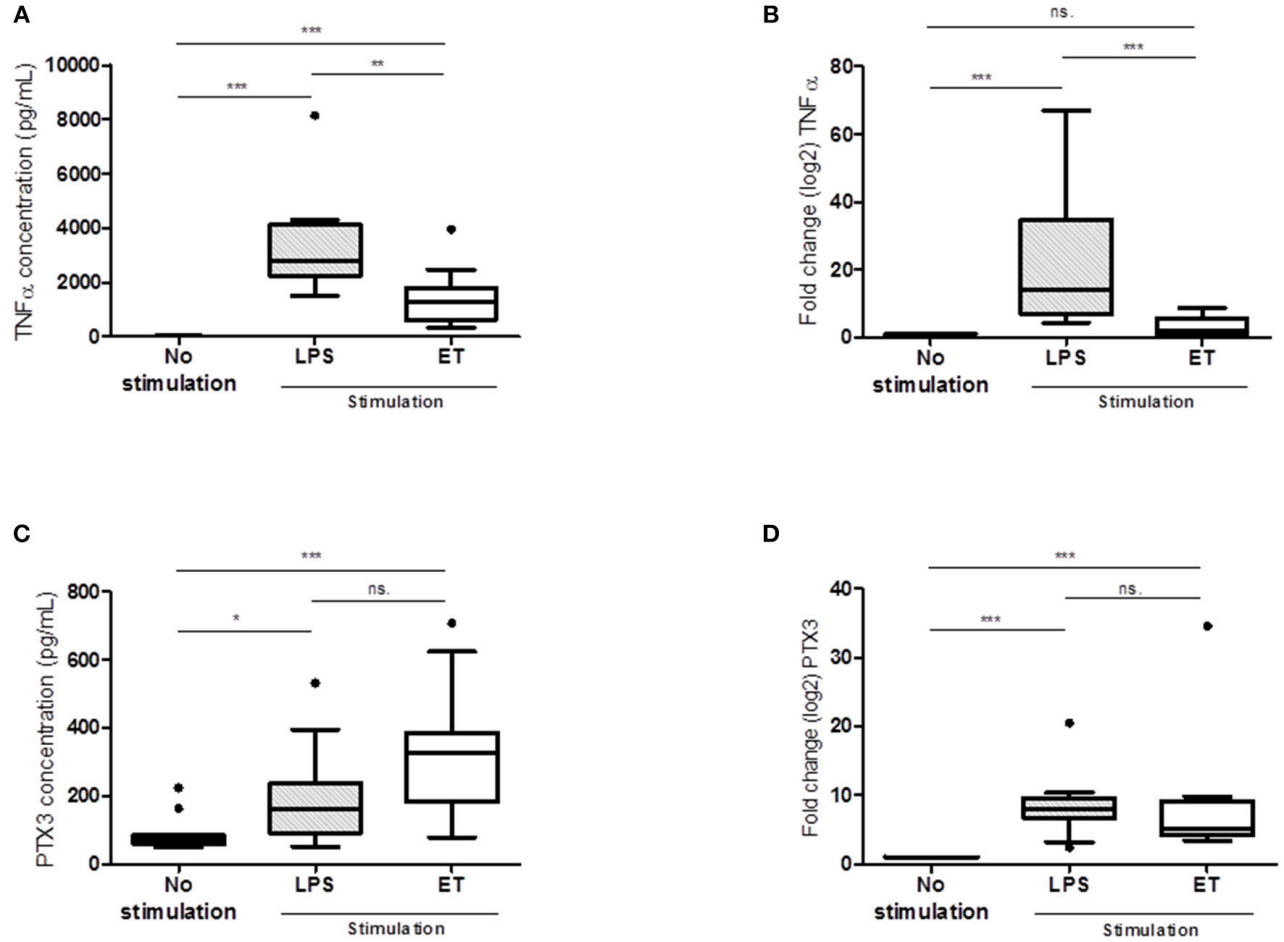
*In vitro* PBMC-based endotoxin tolerance model. LPS condition: stimulation with 100 ng/mL LPS at day 1 and ET (endotoxin tolerance) condition: stimulation with 2 ng/mL LPS at day 0 and 100 ng/mL LPS at day 1. Protein levels **(A–C)** and mRNA gene expression **(B–D)** are plotted for TNFα and PTX3 (*n* = 12). For cytokine expression results are expressed in pg/mL. For mRNA expression results are expressed in fold change. NS: not significant; *p < 0.05; **p < 0.001; ***p < 0.0001.

Given that mature neutrophils are not able to produce *de novo* PTX3 (8), we then hypothesized that the remaining circulating immune cells could be a possible source of PTX3 production during the first days of sepsis. To mimic the altered immune states observed in septic shock patients, healthy neutrophil-free PBMCs (*n* = 12) were stimulated with LPS to reproduce an inflammatory state and a monocyte-anergy state (endotoxin tolerance phenomenon) to study PTX3 behavior. As expected, median TNFα release was two-times lower in the anergy condition (median [IQR]: 1270 [642–1801] pg/mL) than in the inflammatory situation (median [IQR]: 2782 [2257–4124] pg/mL, *p* < 0.001; Figure 4A); a similar result was found at the molecular level (fold change 2 vs. 14 respectively, *p* < 0.0001; Figure 4B). For PTX3, strikingly, there was no significant difference between LPS-induced inflammation (median [IQR]: 160.8 [91.8–238.5] pg/ml) and LPS-induced anergy conditions (median [IQR]: 325.6 [183.1–384.9] pg/mL), although there was a trend toward increased PTX3 secretion in the endotoxin tolerance condition (*p* = 0.07; Figure 4C). A conserved capacity to secrete PTX3 was observed in the *in vitro* model, independently of the inflammatory state. PTX3 gene expression was also not significantly different between both inflammatory *in vitro* conditions (fold change 8 vs. 5 respectively), although there was a trend toward a lower value in the anergy condition (*p* = 0.24, Figure 4D). The trends observed with PBMCs were statistically confirmed with the monocyte THP1-MD2-CD14 cell line (Figure S2).

### mRNA and Protein Decay Analysis

Because of the intriguing behavior of PTX3 mRNA and protein observed in whole blood, in PBMC and in THP1-MD2-CD14 cell line, we sought to investigate PTX3 mRNA half-life and its intracellular protein stability, as well as for TNFα, in inflammation and anergy conditions. Using actinomycin D to inhibit RNA synthesis, we reported that PTX3 mRNA half-life was approximately 4 h, compared to the shorter 0.5 h of TNFα mRNA half-life (Figure S3A). No difference was observed between inflammation and anergy conditions. By adding cycloheximide in cell culture 2 h after LPS stimulation, we observed the behavior of the intracellular content of PTX3 and TNFα in inflammatory and anergy conditions. We noticed that the intracellular content of PTX3 is maintained in the anergy condition compared to a slight decay in LPS-induced inflammation condition. Concerning TNFα, more than 50% of the intracellular content was diminished in both inflammation and anergy conditions only 1 h after the addition of cycloheximide (Figure S3B).

## Discussion

Pentraxin-3, along with other biomarkers such as PCT and lactate, have been stated as clinically informative of disease severity and patient outcome in sepsis and septic shock (24). We confirmed that PTX3 is barely detectable in plasma from HV, around 2 ng/mL in the study reported by Yamasaki et al. (25) and <1 ng/mL herein, while it is much greater in sepsis patients, up to 100 ng/mL in the study reported by Daigo et al. (10) and up to 800-fold greater herein for highest values; this is in accordance with a prompt liberation of preformed PTX3 from granules after an inflammatory trigger (26). We also observed that patients with higher PTX3 plasma levels 3–4 days after ICU admission were those who died during ICU stay. This is in line with the study reported by Huttunen et al. who found that PTX3 values were markedly higher in bacteraemic patients who died compared to survivors (27), also described in a septic shock cohort (11).

In the present study we aimed to assess whether circulating cells could be responsible for the maintenance of PTX3 concentration in the blood of patients over time during severe sepsis. LPS stimulation revealed a state of immunosuppression with a 5-fold lower capacity of patient blood cells to produce TNFα mRNA and release of this cytokine as compared to HV. Strikingly, patient blood cells still had the capacity to secrete PTX3 protein after *ex vivo* LPS stimulation, as did HV. In line with this, the kinetic analysis of PTX3 expression in whole blood stimulated by LPS, both in HV and patients, revealed that the mRNA content is globally decrease from 4 h on, reaching barely detectable levels 24 h post-stimulation whereas the continuous increase in PTX3 protein level reached its maximum at 24 h. The observed delay between the highest mRNA and protein amounts ranged roughly between 20 and 24 h in our conditions. Such observation has already been described in a human fibroblast cell line where such delay in PTX3 synthesis compared to mRNA production was shown (28). Altogether, these results indicate that sustained PTX3 plasma levels in the first week of sepsis could be driven by circulating blood cells, despite their altered immune functions.

Knowing that mature neutrophils are responsible for PTX3 secretion in the first hours of sepsis onset but are unable to produce *de novo* mRNA PTX3, we then aimed to decipher the cellular source of circulating PTX3 neo-synthesis in immunodysregulated septic shock patients. Onset of sepsis is characterized by dysfunctional host response of patients, where an acute hyper-inflammatory phase is established leading to organ damage and hence, early death. Simultaneously, a compensatory response is initiated and can consequently plunge the patient in a longer immunosuppressive phase. To reproduce these altered immune states, a neutrophil-free immune cell compartment was used to evaluate its capacity to produce and release PTX3 in different inflammatory conditions. The endotoxin tolerance model was set up on freshlyPBMC isolated from HV and on the monocyte THP1-MD2-CD14 cell line as well, to mimic an inflammatory- and a tolerant-immune state. At the transcriptional level, PTX3 seems to be a weakly tolerizable gene similar to TNFα, as previously reported in the literature (16, 17). Conversely, increased protein levels observed in anergy condition reflects a non-tolerizable phenotype, different from TNFα but more likely to IL-10, as already described (29). Altogether, these observations, which demonstrate the capacity of PTX3 secretion by tolerizable circulating immune mononuclear cells, emphasize the anti-inflammatory role of PTX3 in sepsis. Of note, PTX3 has been described as associated with IL-10 in atherosclerosis experimental conditions (30) and could help to counteract the pro-inflammatory response observed in sepsis (14). The mechanisms supporting the discordant behavior of PTX3 at transcription and translation levels, different from TNFα, remained to be clearly determined. On one hand, the longer PTX3 mRNA half-life observed *in vitro*−4 vs. 0.5 h for TNFα–may support a sustained protein translation in the tolerant condition. Conversely, it was reported that miRNAs may interact with PTX3 mRNA, making it unstable and responsible for the delay with protein synthesis (28). In addition, we observed a very significantly higher stability of PTX3 protein compared to TNFα, potentially 4 times. Even if we were not able to determine PTX3 protein half-life in our experimental settings, we observed that PTX3 is more stable in the tolerant condition than in the LPS-induced inflammation condition, which would contribute to the higher protein levels in the tolerant condition. Of note, a 2 h PTX3 protein plasma half-life was measured using exogenously administered recombinant protein (31). Interestingly, the absence of difference in mRNA PTX3 half-life between inflammation and tolerant conditions and the apparently higher stability of PTX3 protein in anergy condition would suggest a complex phenomenon involving translation enhancement, although we cannot exclude an increased stability, which could be mediated by protein-protein interaction e.g., complement factors (32). Moreover, PTX3 is described as induced by TNFα (33, 34) but TNFα signaling pathway is known to be disrupted in sepsis (35) which suggests that PTX3 may be induced through other signaling pathways especially if PTX3 has a major anti-inflammatory effect in sepsis (36). This could explain why PTX3 was not altered during sepsis and can still be secreted over the course of the disease. Although this remains to be demonstrated, the PI3K/Akt pathway described in sepsis (37) represents a potential driver of PTX3 expression in sepsis, as recently demonstrated in cancer (38) and in inflammatory condition (39). This pathway, which seems to control the increased IL-1RA anti-inflammatory cytokine in sepsis patients and in LPS-adapted THP1 cells (40), acts indeed by enhancing translation efficacy without interfering with gene transcription. Further experiments are required to evaluate this hypothesis to confirm if sustained circulating PTX3 levels in sepsis are achieved through this signaling pathway.

This study has some limitations to take into consideration regarding the mechanisms of production and action of PTX3 in sepsis pathophysiology. We studied the likely source of plasma PTX3 levels in septic shock patients by immune circulating cells, yet we cannot exclude a role of endothelial cells which was not investigated here (41). Indeed, although our results indicate that plasma PTX3 levels can be explained without endothelial cells contribution, their capacity to secrete PTX3 upon inflammatory signals and the observation that PTX3 protects against histone-mediated endothelial cell cytotoxicity in sepsis (42) and limits the vascular regenerative response (43) deserves further investigation. Moreover, *ex vivo* experiments as well as *in vit*ro models were performed on a limited number of healthy individuals and/or septic patients, and therefore the trends observed require confirmation in larger studies. Results obtained with the monocytic THP1-MD2-CD14 cell line, point out monocytes as (one of) the cell population(s) responsible for such plasma PTX3 source for the first days of sepsis. New pertinent cellular models are required to better understand the contribution of every (blood) cell type to PTX3 expression and the feedback loops with its environment during the host response in sepsis.

In conclusion, circulating PBMCs, despite their immune dysfunctions, could be responsible for the sustained PTX3 plasma levels over the first days of sepsis setting.

## Author Contributions

All authors were involved in the analysis and interpretation of data as well as drafting the manuscript or revising it critically for important intellectual content. CAV and ST-A made substantial contributions to the conception and design of the study. CAV, ST-A and FM designed the experiments. CAV, MM, and MB performed the experiments. TR, FV, VL, GM, KB-P, and BD performed the data collection. CAV, ST-A, MM, FV, and FM performed the data analyses and data interpretation. CAV, FM, and ST-A wrote the paper. CAV, KB-P, TR, FV, MM, VL, GM, BD, AP, FM, and ST-A revised the manuscript content. All authors read and approved the final manuscript. ST-A takes responsibility for the integrity of the data analysis.

### Conflict of Interest Statement

CAV, MM, AP, KB-P and FM are employed by an *in vitro* diagnostic company, bioMérieux. The remaining authors declare that the research was conducted in the absence of any commercial or financial relationships that could be construed as a potential conflict of interest.
