# Supplementary material for: Source of Circulating Pentraxin 3 in Septic Shock Patients
Source: Front Immunol. 2019 Jan 4;9:3048. doi: 10.3389/fimmu.2018.03048 (PMC6338061; doi:10.3389/fimmu.2018.03048)
Supplement: Supplementary file 1 [file Data_Sheet_1.doc]

**Supplementary Table 1.** Nanostring probes A and B for evaluated genes.

| **Target gene** | **Probe A (5’-3’)** | **Probe B (3’-5’)** |
| --- | --- | --- |
| **PTX3** | TCCTCCGGTCTCTCTTATCTCTTCATTGCTAAGAACACTATCCCAGATAT | TCCCCACCCAACAATATTCCCCCGGATGTGACAAGACTCTGC |
| **TNFα** | CAGGCCACACATTCCTGAATCCCAGGTTTCGAAGTGGTGGTCTTGTTGCT | TTCTGGAGGCCCCAGTTTGAATTCTTAGTGGTTGCCAGCACTTCACTGTG |
| **HPRT1** | TGAGCACACAGAGGGCTACAATGTGATGGCCTCCCATCTCCTTCATCACA | CAGTGCTTTGATGTAATCCAGCAGGTCAGCAAAGAATTTATAGCCCCCCT |
| **DECR1** | GGCACCTTTGGTTTTTATAGGCCCTGGTTGAATCACATTGAATCGCATTCC | CTGCCAATCATTTCTTTCTCAAATGTTCCAGTTGGGTCCAGACGGCTAAA |
| **TBP** | GCACGAAGTGCAATGGTCTTTAGGTCAAGTTTACAACCAAGATTCACTGT | TCCTCATGATTACCGCAGCAAACCGCTTGGGATTATATTCGGCGTTTCGG |
