# Supplementary material for: Source of Circulating Pentraxin 3 in Septic Shock Patients
Source: Front Immunol. 2019 Jan 4;9:3048. doi: 10.3389/fimmu.2018.03048 (PMC6338061; doi:10.3389/fimmu.2018.03048)
Supplement: Supplementary file 2 [file Data_Sheet_2.docx]

Supplementary Material

**Source of circulating pentraxin 3 in septic shock patients**

Chloé Albert Vega^1, 2^, Mommert M^1, 2^, Boccard M^1, 3^, Rimmelé T^4, 6^, Venet F^4, 5^, Pachot A^2^, Leray V^6^, Monneret G^4, 5^, Delwarde B^6^, Brengel-Pesce K^1, 2^, Mallet F^1, 2, 4^, Trouillet-Assant S^1, 7, *^.

*** Correspondence:** Dr. Sophie Trouillet-Assant: [sophie.assant@chu-lyon.fr](mailto:sophie.assant@chu-lyon.fr)

**Supplementary Figure S1**

**Supplementary Figure S1.** **Absence of correlation between PTX3 plasma levels and mHLA-DR (D3) of septic shock patients.** Spearman test; correlation coefficient r = -0,317, p = 0,088.

**Supplementary Figure S2**

**
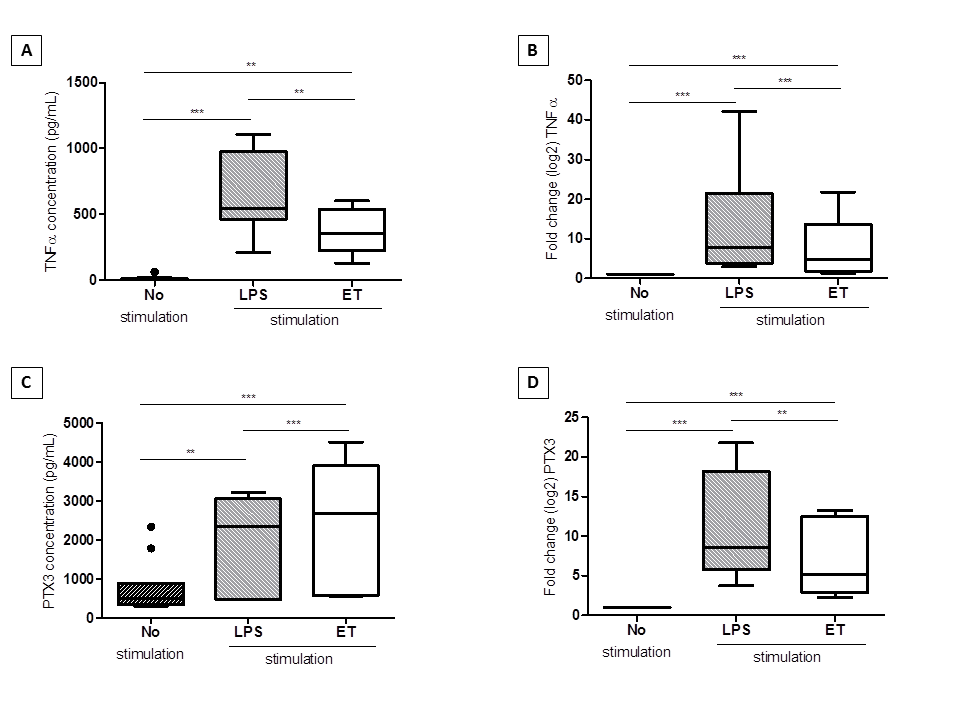
**

**Supplementary Figure S2**. ***In vitro* monocyte THP1-MD2-CD14 cell line-based endotoxin tolerance model**. LPS condition: stimulation with 100 ng/mL LPS at day 1 and ET (endotoxin tolerance) condition: stimulation with 2 ng/mL LPS at day 0 and 100 ng/mL LPS at day 1. Protein levels (A-C) and mRNA gene expression (B-D) are plotted for TNFα and PTX3 (n=11). For cytokine expression results are expressed in pg/mL. For mRNA expression results are expressed in fold change. NS: not significant; **p<0.01; ***p<0.001

**Supplementary Figure S3**


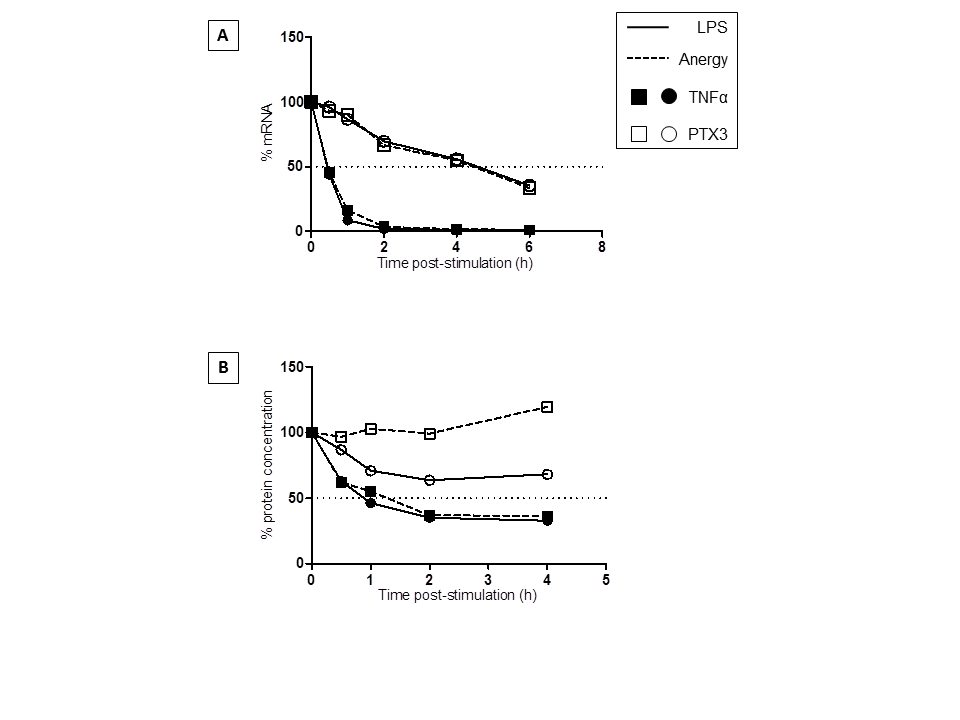


**Supplementary Figure S3.** **PTX3 and TNFα mRNA decay and intracellular protein stability**. Control and tolerant THP1-MD2-CD14 cells were stimulated with LPS (100 ng/mL) for 2 hours. Actinomycin D (5µg/mL) and Cycloheximide (10µg/mL) were then added. A. % of mRNA level of TNFα (filled symbols) and PTX3 (open symbols) in LPS-induced inflammation (solid line) and in LPS-induced anergy (dashed line) in THP1-MD2-CD14 cells. B. % of intracellular protein level of TNFα (filled symbols) and PTX3 (open symbols) in LPS-induced inflammation (solid line) and in LPS-induced anergy (dashed line) in THP1-MD2-CD14 cells. mRNA and intracellular protein levels were expressed as a percentage of the maximal mRNA and protein level, respectively.
